# Reconstruction survival of segmental megaendoprostheses: a retrospective analysis of 28 patients treated for intercalary bone defects after musculoskeletal tumor resections

**DOI:** 10.1007/s00402-020-03583-4

**Published:** 2020-08-29

**Authors:** Arne Streitbürger, Jendrik Hardes, Markus Nottrott, Wiebke K. Guder

**Affiliations:** 1 Department of General and Tumor Orthopedics, University Hospital Muenster, Albert-Schweitzer-Campus 1, Building A1, 48149 Muenster, Germany; 2 Department of Orthopedic Oncology, University Hospital Essen, Hufelandstrasse 55, 45147 Essen, Germany

**Keywords:** Intercalary endoprosthetic reconstruction, Segmental prosthesis, Aseptic stem loosening, Megaendoprostheses

## Abstract

**Introduction:**

Intercalary endoprosthetic reconstructions have been reserved for patients with a limited life expectancy due to reports of high rates of early mechanical and reconstruction failure.

**Materials and methods:**

In this study, we retrospectively analyzed 28 patients who underwent intercalary endoprosthetic reconstruction of the femur (*n* = 17) or tibia (*n* = 11) regarding reconstruction survival and causes of complications and reconstruction failure.

**Results:**

A total of 56 stems were implanted in this collective, 67.9% of which were implanted using cementation. Eight different stem designs were implanted. The mean patient age at the time of operation was 42.3 years. The mean bone defect needing reconstruction measured 18.5 cm. Resection margins were clear in 96.4% of patients. Of twenty-six complications, five were not implant-associated. We observed infection in 10.7% (*n* = 3) and traumatic periprosthetic fracture in 3.6% (*n* = 1) of cases. The most frequent complication was aseptic stem loosening (ASL) (53.8%; *n* = 14) occurring in eight patients (28.6%). The metaphyseal and meta-diaphyseal regions of femur and tibia were most susceptible to ASL with a rate of 39.1% and 31.3% respectively. No ASLs occurred in epiphyseal or diaphyseal location. Overall reconstruction survival was 43.9% and 64.3% including patients who died of disease with their implant intact. Overall limb survival was 72.7%.

**Conclusions:**

Proper planning of segmental reconstructions including stem design with regard to unique anatomical and biomechanical properties is mandatory to address the high rates of ASL in metaphyseal and metadiaphyseal stem sites. With continued efforts of improving stem design in these implantation sites and decreasing rates of mechanical failure, indications for segmental megaendoprostheses may also extend to younger patients with the localized disease for their advantages of early weight bearing and a lack of donor-site morbidity.

## Introduction

After intercalary tumor resections, surgeons still prefer biological reconstructions of these defects to megaendoprosthetic ones [1]. Biological reconstruction techniques include the use of allografts, (irradiated) autografts, (vascularized) fibula grafts, as well as segmental transport (callotasis/bone transport) and tissue engineering strategies (i.e. Masquelet’s technique). They are often reinforced by compound osteosyntheses, including plating and intramedullary nailing [2, 3]. Yet, complication rates of these reconstructions are high and include pseudarthrosis, non-union, fracture, infection, and generally longer periods of non-weight bearing and use of orthoses [4, 5]. Therefore, they may not be the best option in patients undergoing chemotherapy, elderly patients and those with a limited life expectancy.

Despite advantages such as early mobility with full weight bearing, joint and growth plate preservation and a lack of donor-site morbidity, indications for the use of segmental megaendoprostheses have been limited to (elderly) patients with metastatic disease and severe pain or instability [1, 2]. The main reason for this cautious behavior towards segmental megaendoprostheses is reports of high rates of early mechanical failure [2], leading to reconstruction failure and subsequently a lower rate of permanent limb salvage. Aseptic loosening followed by implant wear and breakage is reported to be the most common complication [1]. While Fuchs et al. report that an adequate amount of cortical bone stock and approximately 5 cm of intramedullary canal are required for implantation of standard stems, they also state that the risk of stress shielding and aseptic loosening increases with stem length [2].

Since stem site varies from that of standard stems used for osteoarticular endoprostheses in the diaphysis and different biomechanical conditions apply (i.e. the absence of a joint, which poses an outlet for shear forces, may lead to additional strain on stems in intercalary reconstructions), different stem properties and designs may be necessary to address higher rates of mechanical failure observed so far.

Therefore, the purpose of this study was to analyze implant survival and causes for implant failure in 28 patients treated with segmental megaendoprostheses after intercalary bone and soft tissue tumor resections of the lower limb. Due to the considerations mentioned above and aiming at joint preservation despite little remaining bone stock, we implanted a number of custom-made stems using alternative designs and will present the outcomes achieved with these compared to conventional stem designs.

## Material and methods

Twenty-eight patients, treated between 1999 and 2017, were identified to have undergone either intercalary femoral (*n* = 17) or tibial (*n* = 11) endoprosthetic reconstruction using a modular megaendoprosthetic implant following segmental tumor resection. Operations were performed by four senior orthopedic surgeons with a specialty in orthopedic oncology at the Department of General and Tumor Orthopedics, Muenster, Germany.

Patient data were collected from archived patient files of the orthopedic department in an anonymized fashion.

### Patient characteristics

Patient age at the time of operation was a mean of 42.3 years (range 10–80 years) for both locations and 47.1 years (range 10–80 years) for femoral, 38.4 years (12–59 years) for tibial segmental resections. In all patients (*n* = 28), incisional biopsy was performed to confirm the diagnosis prior to tumor resection. Seven patients with a femoral tumor resection had primary tumors located in the femur, ten patients in the soft tissue surrounding the femur. All patients (*n* = 11) with tibial tumor resections were treated for primary bone tumors of the tibia. Histological diagnoses separated for femoral bone and soft tissue tumors follow in decreasing order: bone: osteosarcoma (*n* = 2), kidney cancer metastasis (*n* = 2), Ewing sarcoma (*n* = 1), chondrosarcoma (*n* = 1) and breast cancer metastasis (*n* = 1). Soft tissue: Sarcoma not otherwise specified (NOS) (*n* = 6), myxofibrosarcoma (*n* = 2), extraskeletal PNET (primitive neuroectodermal tumor) (*n* = 1), extraskeletal chondrosarcoma (*n* = 1). In tibial segmental resections, histological diagnoses were as follows in decreasing order: bone: NOS (*n* = 4), Ewing sarcoma (*n* = 3), osteosarcoma (*n* = 2), adamantinoma (*n* = 1) and kidney cancer metastasis (*n* = 1). Tumor size at the time of operation was > 5 cm in the longest diameter in ten patients and > 10 cm in the longest diameter in seventeen patients. In one patient, information on tumor size was not available. Seven patients had primary operations elsewhere prior to segmental tumor resection for recurrent or persistent tumors at our department. A more detailed report of patient characteristics can be seen in Tables 1 and 2.

**Table 1:** Patient characteristics—femur

No.	Age	Diagnosis	Grading	Bone/ST	Size	Enneking classification	Previous operation	Chemo	Response	Radiation	FU
Femur/Thigh											
1	20	Extraskeletal chondrosarcoma	3	ST	> 10	IIa		Y	5–6	N	DOD 11
2	10	Ewing	3	Bone	5–10	IIb		Y	2	N	NED 111
3	32	Extraskeletal PNET	3	ST	5–10	IIa		Y	1	N	NED 95
4	62	Chondrosarcoma	2	Bone	Na	IIa	Intralesional curettage	Y		N	NED 217
5	80	NOS	2–3	Bone	5–10	IIb		N		Y	DOD 4
6	49	Breast Cancer Metastasis		Bone	> 10	Met		Y		Y	DOD 139
7	48	Myxofibrosarcoma	3	ST	5–10	IIa	Intralesional resection	N		Y	NED 119
8	55	NOS	3	ST	> 10	IIa	Intralesional resection	Y		N	DOD Na
9	60	Kidney Cancer Metastasis		Bone	5–10	Met		Y		Y	AWD 76
10	41	NOS	3	ST	> 10	IIa	Intralesional resection	Y		Y	NED 71
11	35	NOS	3	ST	> 10	IIa		Y		Y	AWD 43
12	54	NOS	3	ST	> 10	IIa		Y		Y	DOD 42
13	63	Osteosarcoma	3	Bone	> 10	IIb		Y	2	N	NED 60
14	62	Kidney Cancer Metastasis		Bone	5–10	Met	Intramedullary nailing	Y		Y	NED 67
15	53	NOS	3	ST	> 10	IIa	Marginal resection	N		Y	NED 63
16	60	Myxofibrosarcoma	1–2	ST	> 10	IIa		N		Y	AWD 64
17	17	Osteosarcoma	3	Bone	> 10	IIb		Y	2	N	NED 10

*No*. number, *Age* at diagnosis (years), *PNET* primitive neuroectodermal tumor, *NOS* Sarcoma not otherwise specified, *ST* soft tissue, *Size* in cm, *Na* not available, *IIa* intracompartmental, *IIb* extracompartmental, *Y* yes, *N* no, *Response* to chemotherapy according to Salzer–Kuntschik, *FU* follow-up in months, *DOD* death of disease, *NED* no evidence of disease, *AWD* alive with disease

**Table 2:** Patient characteristics—tibia

No.	Age	Diagnosis	Grading	Bone/ST	Size	Enneking classification	Previous operation	Chemo	Response	Radiation	FU
Tibia											
1	17	Osteosarcoma	3	Bone	> 10	IIa		Y	4	N	DOD 31
2	44	Adamantinoma	1	Bone	5–10	IIb		N		N	NED 81
3	26	Ewing	3	Bone	5–10	IIb		Y	1	N	NED 81
4	44	NOS	2–3	Bone	5–10	IIb		Y	2	N	DOD 32
5	47	NOS	G3	Bone	> 10	IIb		N		N	NED 78
6	58	NOS	2	Bone	5–10	IIa		Y		N	NED 119
7	59	NOS	3	Bone	> 10	IIb	Intralesional Curettage	Y	3	N	DOD 19
8	59	Kidney Cancer Metastasis		Bone	> 10	Met		Y		Y	AWD 41
9	18	Osteosarcoma	3	Bone	> 10	IIb		N		N	NED 57
10	12	Ewing	3	Bone	> 10	IIb		Y	1	N	NED 51
11	17	Ewing	3	Bone	> 10	IIa		Y	1	N	NED 3

*No*. number, *Age* at diagnosis (years), *PNET* primitive neuroectodermal tumor, *NOS* Sarcoma not otherwise specified, *ST* soft tissue, *Size* in cm, *IIa* intracompartmental, *IIb* extracompartmental, *Y* yes, *N* no, *Response* to chemotherapy according to Salzer–Kuntschik, *FU* follow-up in months, *DOD* death of disease, *NED* no evidence of disease, *AWD* alive with disease

**Fig. 1:**
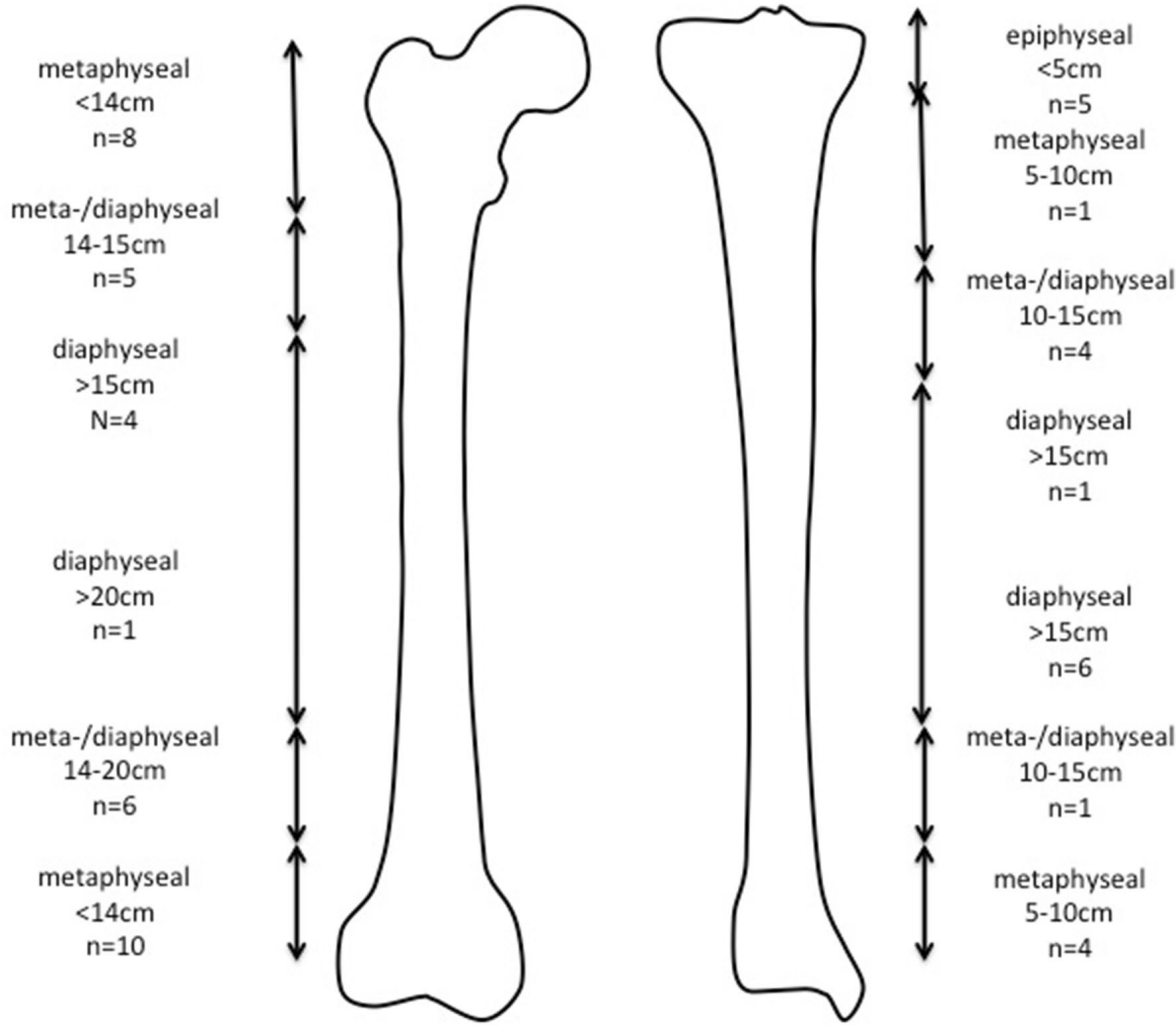
Femoral and tibial osteotomy levels

In 28 patients, a total of 56 stems were implanted after segmental tumor resections of the femur (*n* = 17; 34 stems) and tibia (*n* = 11; 22 stems). Osteotomy levels were measured by distance from the adjacent joint in cm. The extent of remaining bone after intercalary tumor resection was sorted into four different anatomical categories (epiphyseal, metaphyseal, meta-diaphyseal and diaphyseal) and is depicted in Fig. 1. Cementation was performed in 67.9% of stems (*n* = 38) using polymethyl methacrylate (i.e. Heraeus Medical PALACOS^®^), while 32.1% of stems (*n* = 18) were implanted in a non-cemented fashion. The type of stem fixation was planned prior to operation. Cementation was indicated in elderly patients without the ability for periodic partial weight bearing and/or comorbidities significantly affecting bone viability (i.e. smoking history, cardiovascular disease, diabetes mellitus, prior operation with a negative impact on bone viability as assessed in preoperative imaging, the necessity for adjuvant radiation therapy). Cementless fixation was preferred in younger patients without relevant mobility restrictions and/or comorbidities. Chemotherapy did not affect the type of stem fixation chosen. Use of modular off-the-shelf diaphyseal implants necessitated cementation due to stem design regardless of patient characteristics. More detailed information on cementation, type of implanted stem and additional implantation of locking screws is listed in Tables 3 and 4. The mean resection length and resulting bone defect was 18.5 cm (range 10–29 cm).

**Table 3:** Reconstruction details—femur

No.	PO	Site	Prox. stem	DO	Site	Distal stem	Res length	Rev	No. rev	Cause revision	Stem affected	Procedure	Time to revision	Rec. failure	Rec. survival
Femur/thigh															
1	12	M	FS NC	14	MD	DI C 2xI	24	N	–	–	–	–	–	–	DOD 11
2	4.5	M	CMS NC 1xI	7.5	*M*	*CMS C 1xI*	20	Y	3	ASL 2x	Distal 2x	1× stem change 1× distal femur	16/94	94	–
3	19	D	FS NC	12	*M*	*DI C*	17	N	–	ASL (18 mo.)	Distal	None	–	–	95
4	Na	MD	FS C	Na	M	DI C 2xI	22	Y	1	LR	–	Hip disarticulation SLP	11	11	–
5	13	M	DI C 2xI	18	MD	DI C 2xI	21	N	–	–	–	–		–	DOD 4
6	Na	M	DI C	Na	M	DI C	16	Y	2	Infection	–	Two–stage revision	6	–	DOD 139
7	10	M	DI C 2xI	10	M	CMS C 2xI	28	Y	1	WHD	–	Soft tissue revision	5	–	119
8	23	D	FS C	11	M	DI C 2xI	12	Y	1	LR	–	Hip disarticulation	10	10	–
9	15	MD	DI C 2xI	26	D	FS C	10	Y	2	Periprosthetic fracture	Prox	Prox. Femur	15	15	–
10	21	D	FS NC	17	MD	DI C 2xI	15	Y	5	Compartment syndrome	–	Knee disarticulation	–	–	71
11	14	MD	TS C	11	MD	DI C 2xI	16	N	–	–	–	–	–	–	43
12	18	D	FS C	10	M	DI C 2xI	17	N	–	–	–	–	–	–	DOD 42
13	15	*MD*	*TS C*	8	*M*	*CMS C 2xI*	29	Y	2	ASL 3x	Distal 1x Prox. 2x	2× stem change prox., 1× distal femur	6/41	6	–
14	13	M	DI C 2xI	16	MD	FS C	13	N	–	–	–	–	–	–	67
15	15	*MD*	*TS C*	13	*M*	*DI C 2xI*	21	Y	2	ASL 2x	Distal 1x Prox. 1x	1× stem change, 1× distal femur	9/18	18	–
16	10	M	CMS NC 1xI	19	MD	DI C 2xI	16	N	–	–	–	–	–	–	64
17	9	M	CMS NC 1xI	10	M	CMS NC	25	N	–	–	–	–	–	–	10

*No.* Number, *PO* proximal osteotomy (cm below great trochanter), *D* diaphyseal, *M* metaphyseal, *MD* meta-diaphyseal, *FS* modular standard femoral stem (curved, 12 cm length), *NC* cementless, *CMS* custom-made stem, *C* cemented, *DI* modular stem (straight, 10 cm length), *I* stem-locking screw, *TS* tibial stem (straight, 12 cm length), *DO* distal osteotomy (cm above knee joint), *Res*. *Length* resection length, *Rev.* revision surgery, *N* no, *Y* yes, *No*. *Rev.* number of revision operations, *ASL* aseptic stem loosening (given in italics), *LR* local recurrence, *WHD* wound healing disorder, *prox.* proximal, *SLP* stump lengthening procedure, *Time to revision* in months, *Rec.* Reconstruction, *DOD* death of disease

**Table 4:** Reconstruction details—tibia

No.	PO	Site	prox. stem	DO	Site	Distal stem	Res length	Rev	No. rev	Cause revision	Stem affected	Procedure	Time to revision	Rec. failure	Rec. survival
Tibia															
1	2.5	E	CMS NC 5xI	16	D	TS NC	24	Y	1	LR + WHD	–	Knee disarticulation	–	12	–
2	15	D	TS C	5	*M*	*CMS NC 2xI*	16	Y	3	ASL 2x + WHD	Distal 2x	Stem change 1x, below–knee amputation	8/56	56	–
3	14	MD	TS C	6	*M*	*CMS NC 2xI*	18	Y	3	ASL	Distal	Stem change	14	–	81
4	14	*MD*	*DI C*	6	M	CMS NC 2xI	19.5	Y	1	ASL 2x	Prox	stem change 1x, none 1x	19	–	DOD 32
5	12	MD	DI C	10	MD	DI C	13	Y	7	Infection/soft tissue def	–	Below-knee amputation	–	2	–
6	3	E	CMS NC 5xI	21	D	TS C	17	N	–	–	–	–	–	–	119
7	11	MD	CMS DI C 2xI	7	M	CMS C 2xI	25	Y	1	WHD	–	Soft tissue revision	–	–	DOD 19
8	11	*M*	*DI C 2xI*	16	D	TS C	15	Y	5	ASL + LR (1) Infection (2)	Prox	Prox. Tibia (1) None (2)	32	32	–
9	3.5	E	CMS NC 5xI	29	D	TS NC	14	N	–	–	–	–	–	–	57
10	3.5	E	CMS NC 5xI	19	D	TS NC	17	Y	1	WHD	–	Soft tissue revision	–	–	51
11	2.5	E	CMS NC 4xI	20	D	TS NC	18	N	–	–	–	–	–	–	3

*No.* Number, *PO* proximal osteotomy (cm below great trochanter), *E* epiphyseal, *D* diaphyseal, *M* metaphyseal, *MD* meta-diaphyseal, *NC* cementless, *CMS* custom-made stem, *C* cemented, *DI* modular stem (straight, 10 cm length), *I* stem-locking screw, *TS* tibial stem (straight, 12 cm length), *DO* distal osteotomy (cm above knee joint), *Res.* *length* resection length, *Rev.* revision surgery, *N* no, *Y* yes, *No*. *Rev*. number of revision operations, *ASL* aseptic stem loosening (given in italics), *LR* local recurrence, *def*. deficiency, *WHD* wound healing disorder, *prox*.: proximal, *Time to revision* in months, *Rec*. Reconstruction, *DOD* death of disease

Resection margins were wide in twenty-five, marginal in two and intralesional in one patient. Local recurrence occurred in four patients (femur/thigh *n* = 2; tibia *n* = 2), two of which had primary operations elsewhere prior to segmental tumor resection and in one patient with osteosarcoma who discontinued adjuvant chemotherapy after one cycle.

Local radiation therapy was administered in eleven patients, ten of which had femoral and one a tibial segmental reconstruction. A total of twenty-one patients underwent chemotherapy (femoral *n* = 13; tibial *n* = 8).

Sixteen patients are currently alive with no evidence of disease (NED), four patients are alive with disease (AWD) and eight patients died of disease (DOD) after a mean time of 35 months after operation (range 4–139 months). The mean current follow-up of living patients is 75 months (range 3–217 months).

### Stem properties

A total of eight different stem designs were used. Custom-made stems were planned using plain radiographs or preoperative computer tomography scans (DICOM format, reconstruction matrix 512 × 512, slice thickness ≤ 1 mm).

**Fig. 2:**
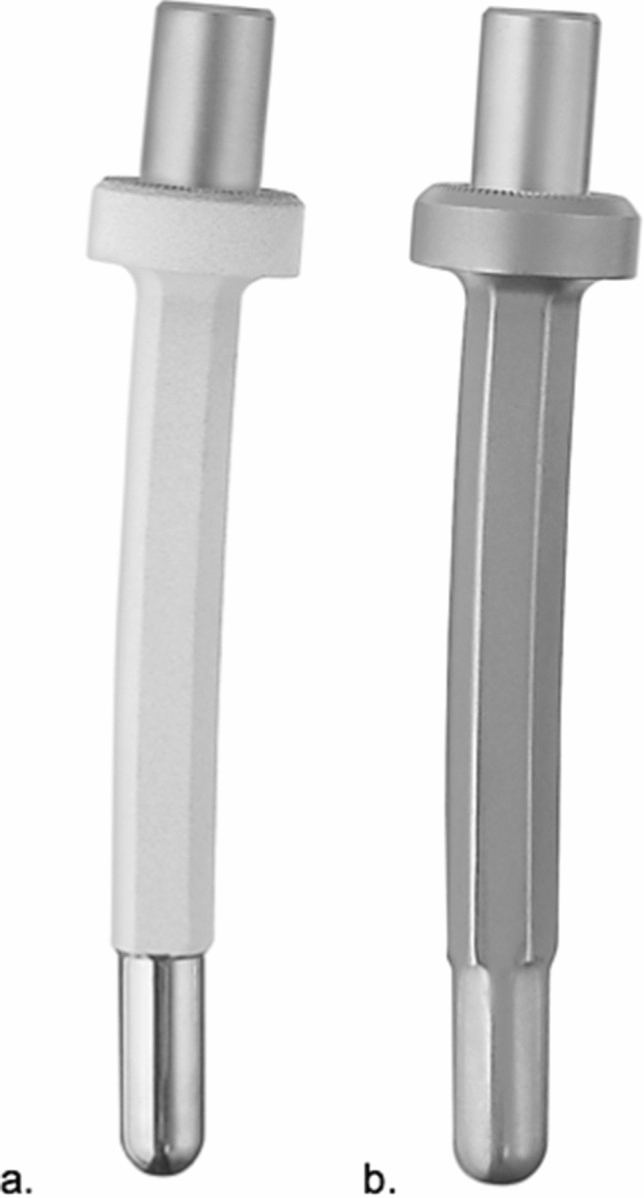
Modular conventional femoral stem (**a** hydroxyapatite surface, **b** smooth surface) (implantcast, Buxtehude, Germany)
  Off-the-shelf modular femoral stem: length 120 mm, curved, tapered. Non-cemented fixation: 12–18 mm diameter, titanium–aluminum–vanadium alloy (ISO 5832-3), rough surface with hydroxyapatite coating. Cemented fixation: 11–17 mm diameter, cobalt–chrome–molybdenum alloy (ISO 5832-4), smooth surface. Solid, hexagonal stem design. Indication: Diaphyseal and metadiaphyseal femur.

**Fig. 3:**
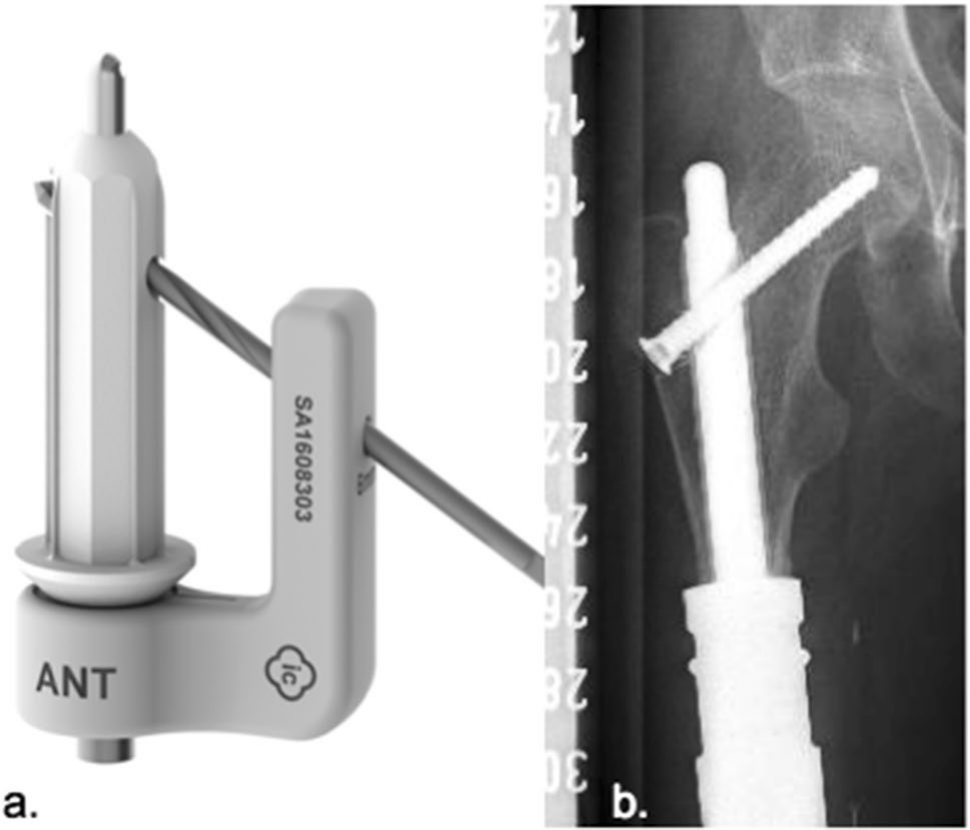
Custom-made modular short proximal femoral stem (**a** Buxtehude stem with PSI (patient-specific implant) template for pre-drilling the femoral head screw; **b** plain radiograph of an implanted Buxtehude stem) (implantcast, Buxtehude, Germany)
  Custom-made modular femoral stem: length and diameter adjustable depending on remaining bone stock, tapered. Non-cemented fixation: titanium–aluminum–vanadium alloy (ISO 5832-3), rough surface with hydroxyapatite coating. Tricalcium phosphate (TCP) coating is also possible but was not used in this collective. Solid, hexagonal stem with protruding fins. Implantation of a femoral head screw is optional. Indication: Metaphyseal femur.
  Dieckmann et al. first reported stem design in 2014 [6].

**Fig. 4:**
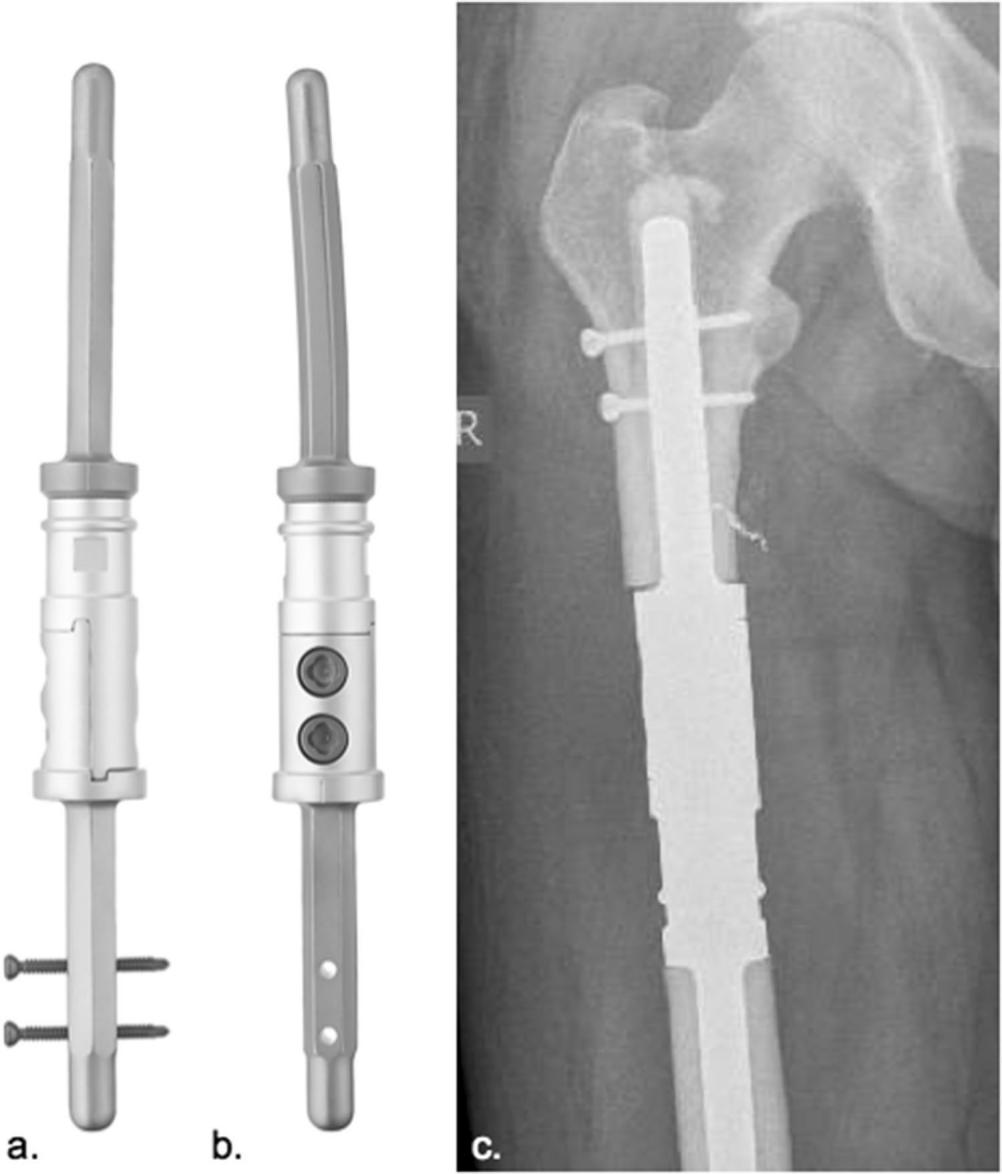
Modular diaphyseal Implant stem (**a** a.p. view of implant; **b** lateral view of implant; **c** a.p. radiograph of a diaphyseal implant used in the proximal femur) (implantcast, Buxtehude, Germany)
  Off-the-shelf modular diaphyseal implant (DI) including 100 mm stem, tapered. Cemented fixation: 15, 17, 19 mm diameter, cobalt–chrome–molybdenum alloy (ISO 5832-4), smooth surface. Solid, hexagonal stem design. Implantation of two additional locking screws is optional. Stem is manufactured in one piece with a half-body of the segmental prosthesis and may be connected with the prosthetic body using a 100 or 120 mm connection piece. Indication: Diaphyseal, metadiaphyseal femur.

**Fig. 5:**
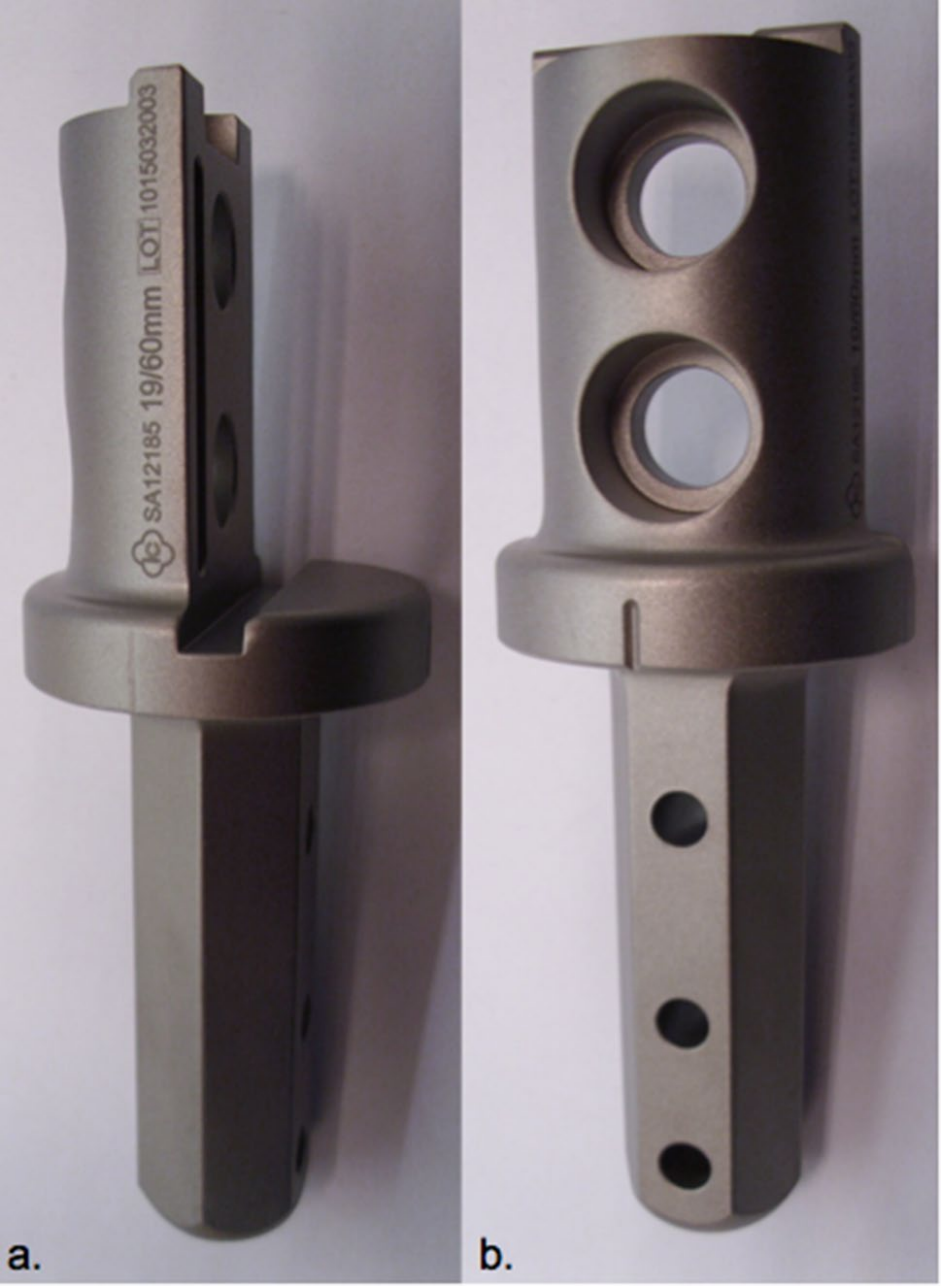
Custom-made modular short distal femur stem (**a** a.p. and **b** lateral view of implant) (implantcast, Buxtehude, Germany)
  Custom-made modular distal femoral stem: length and diameter adjustable depending on remaining bone stock. Cemented fixation: cobalt–chrome–molybdenum alloy (ISO 5832-4), smooth surface. Stem is manufactured like a diaphyseal implant and comes in one piece with a half-body of the segmental prosthesis and the same connection options and option of two interlocking screws (see 3.). Indication: metaphyseal distal femur.

**Fig. 6:**
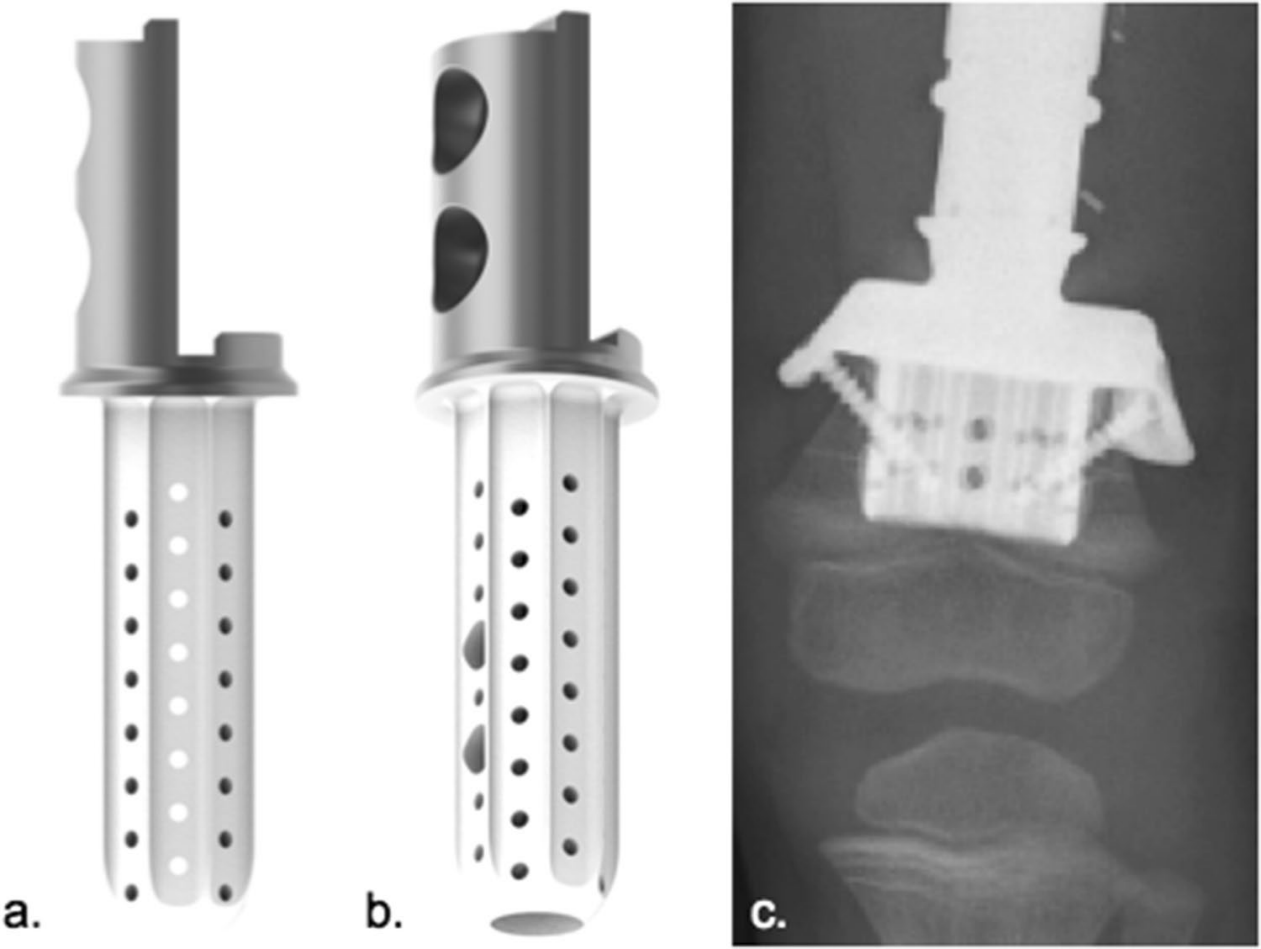
Custom-made hollow distal femur stem (**a**, **b** a.p. and oblique view of implant; **c** a.p. view of implanted stem with double locking screws and external implant flaps increasing contact surface) (implantcast, Buxtehude, Germany)
  Custom-made modular hollow distal femur stem: length and diameter adjustable depending on remaining bone stock. Non-cemented fixation: titanium–aluminum–vanadium alloy (ISO 5832-3), rough surface with hydroxyapatite coating (TCP also possible). Hollow, hexagonal stem design with protruding fins and option of implantation of two interlocking screws. Indication: Metadiaphyseal and metaphyseal distal femur.

**Fig. 7:**
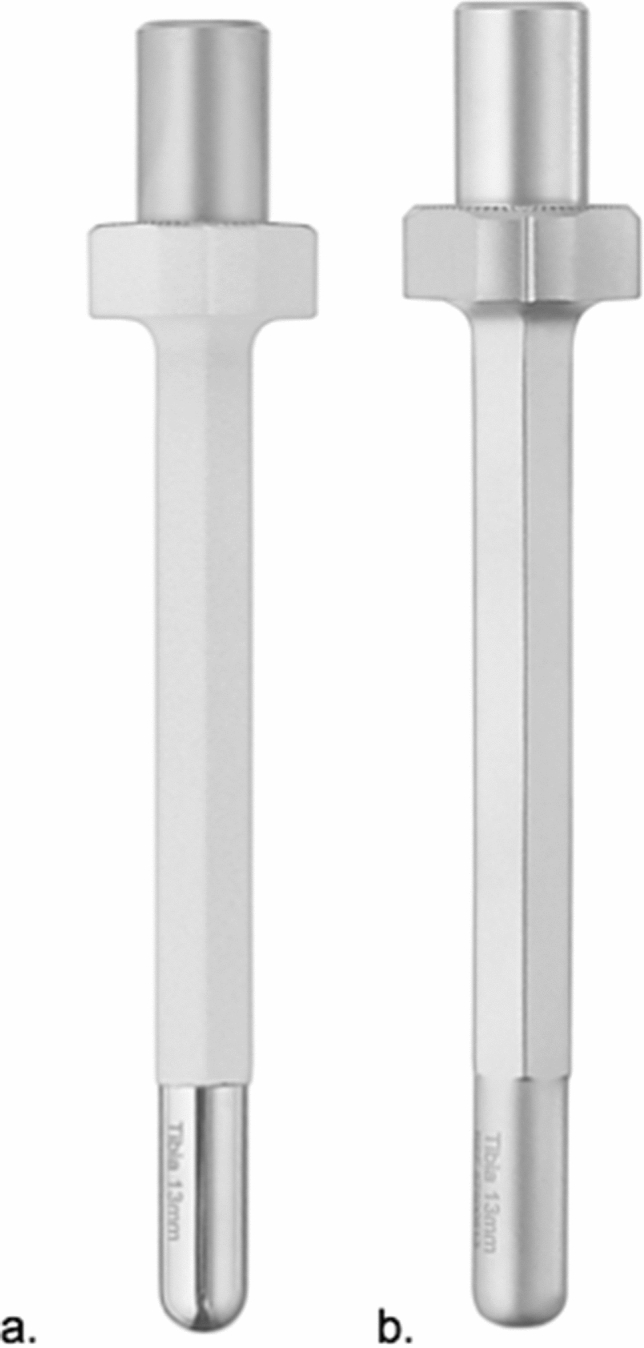
Modular conventional tibial stem (**a** hydroxyapatite surface, **b**.smooth surface) (implantcast, Buxtehude, Germany)
  Off-the-shelf modular tibia stem: length 120 mm, straight, tapered. Non-cemented fixation: 12–16 mm diameter, titanium–aluminum–vanadium alloy (ISO 5832-3), rough surface with hydroxyapatite coating. Cemented fixation: 11–15 mm diameter, cobalt–chrome–molybdenum alloy (ISO 5832-4), smooth surface. Solid, hexagonal stem design. Indication: Diaphyseal tibia.

**Fig. 8:**
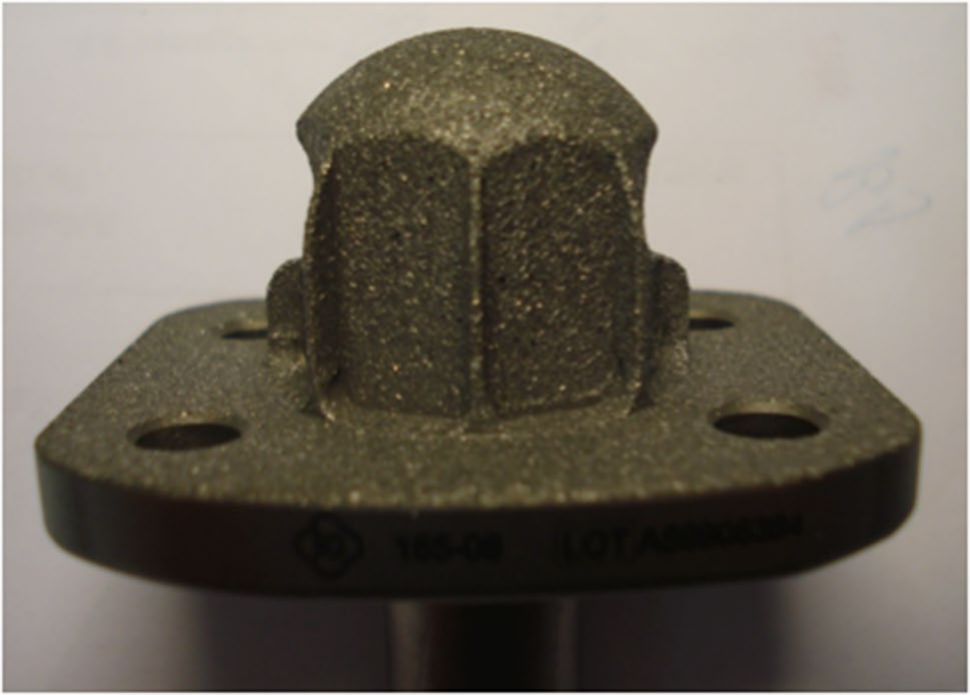
Custom-made modular ultra-short proximal tibia stem (implantcast, Buxtehude, Germany)
  Custom-made modular ultra-short proximal tibia stem: length and diameter adjustable depending on remaining bone stock. Non-cemented fixation: titanium–aluminum–vanadium alloy (ISO 5832-3), rough surface with hydroxyapatite coating (TCP also possible). Solid or hollow, hexagonal stem design with protruding fins and option of implantation of interlocking screw. Indication: epi- and metaphyseal proximal tibia. This stem design was first reported in 2017 [7].

**Fig. 9:**
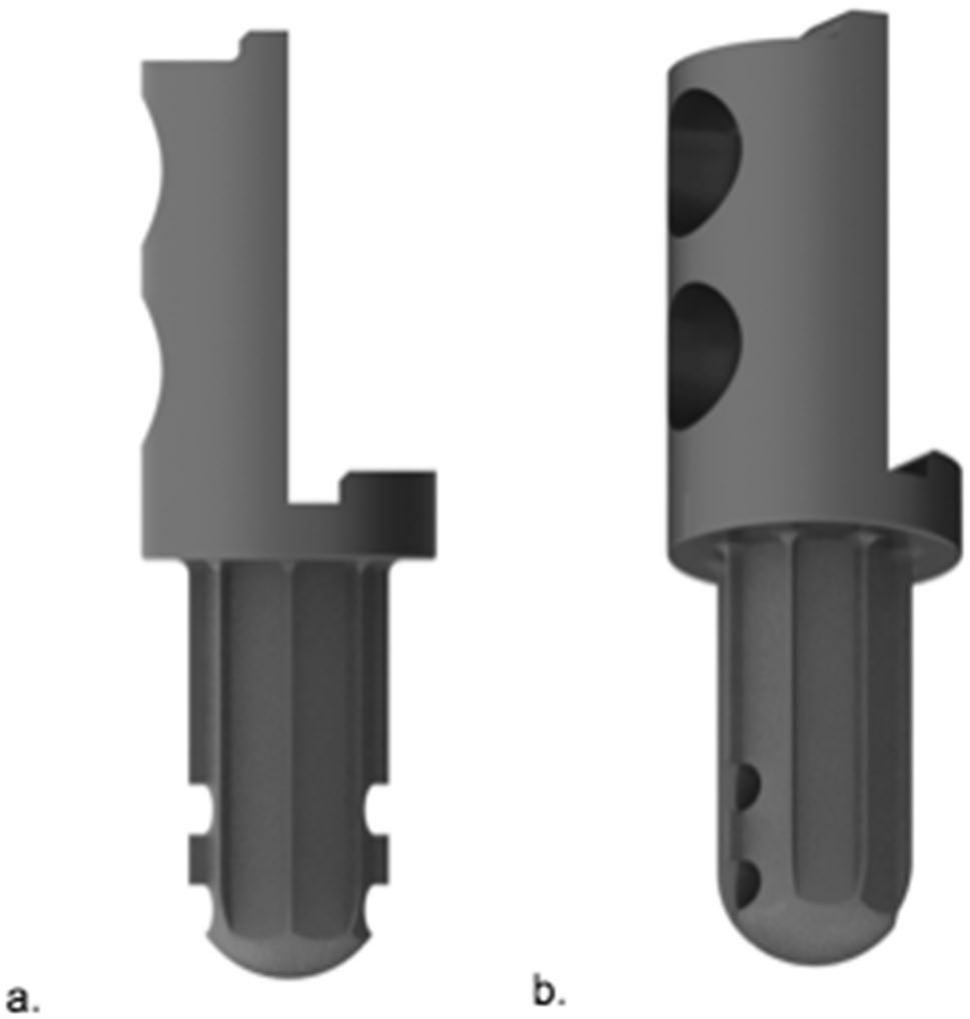
Custom-made modular distal tibia stem (**a** a.p. view and **b** lateral view of implant) (implantcast, Buxtehude, Germany)
  Custom-made modular, short distal tibia stem: length and diameter adjustable depending on remaining bone stock. Non-cemented fixation: titanium–aluminum–vanadium alloy (ISO 5832-3), rough surface with hydroxyapatite coating (TCP also possible). Cemented fixation: cobalt–chrome–molybdenum alloy (ISO 5832-4), smooth surface. Stem is manufactured like a diaphyseal implant and comes in one piece with a half-body of the segmental prosthesis and the same connection options and option of two interlocking screws (see 3.). Indication: Epi- and metaphyseal distal tibia.

1. MUTARS^®^ femoral stem non-cemented/cemented (implantcast, Buxtehude, Germany) (Fig. 2a, b)

2. MUTARS^®^ custom-made short proximal femur stem (Buxtehude stem) (implantcast, Buxtehude, Germany) (Fig. 3a, b).

3. MUTARS^®^ diaphyseal implant stem (implantcast, Buxtehude, Germany) (Fig. 4a–c)

4. MUTARS^®^ custom-made short distal femur stem (implantcast, Buxtehude, Germany) (Fig. 5a, b)

5. MUTARS^®^ custom-made hollow-distal femur stem (implantcast, Buxtehude, Germany) (Fig. 6a–c)

6. MUTARS^®^ tibia stem non-cemented/cemented (implantcast, Buxtehude, Germany) (Fig. 7 a/b)

7. MUTARS^®^ custom-made ultra-short proximal tibia stem (implantcast, Buxtehude, Germany) (Fig. 8)

8. MUTARS^®^ custom-made ultra-short distal tibia stem (implantcast, Buxtehude, Germany) (Fig. 9a, b)

### Complications

Complications, which resulted in surgical intervention, were categorized according to the Henderson classification [8].

### Reconstruction survival/failure

Reconstruction survival was defined as preservation of the intercalary reconstruction and adjacent joints disregarding whether part of the implant had to be replaced in revision surgery. The necessity of amputation above the segmental resection or a conversion toward an osteoarticular reconstruction was defined as reconstruction failure.

### Statistical analysis

Statistical analysis was performed using the Kaplan Meier estimation and Cox regression to analyze overall implant and reconstruction survival. Univariate analysis was used to analyze and compare single influencing parameters. A *p* value of < 0.05 was considered as statistically significant.

### Compliance with ethical standards

#### Research involving human participants

All procedures performed in studies involving human participants were in accordance with the ethical standards of the institutional and/or national research committee and with the 1964 Helsinki declaration and its later amendments or comparable ethical standards. For this study, formal consent was obtained from the local ethical committee (Reference number 18-8469-BO). This article does not contain any studies with animals performed by any of the authors.

#### Informed consent

Informed consent was obtained from all individual participants included in the study.

## Results

In this collective, we observed a total of twenty-six complications (femoral *n* = 13, tibial *n* = 13). A detailed overview of stem and operation site properties as well as complications is presented in Table 3 and 4*.*

### Mechanical complications

#### Type 1—Soft tissue failure

Five cases of soft tissue failure (femoral *n* = 1, tibial *n* = 4) were observed. Wound healing disorders without the involvement of the implant were treated successfully by revision operation in all five cases.

Four patients were treated by a minor superficial debridement and secondary suture, while one patient had to undergo transplantation of a vascularized latissimus muscle flap for a persistent wound healing disorder without the involvement of the reconstruction.

In one case, compartment syndrome following a segmental tumor resection in the thigh including the femoral vessels was reconstructed using a vascular femoral graft. Postoperative vascular occlusion of the graft lead to ischemia and a compartment syndrome of the lower leg and had to be treated by knee disarticulation, leaving the femoral diaphyseal reconstruction intact.

#### Type 2—Aseptic loosening

The most frequent problem observed was aseptic stem loosening (ASL). A total of fourteen ASLs (53.8%; *n* = 14/26) were observed in eight patients (femoral *n* = 4; tibial *n* = 4) (28.6%). One patient had a synchronous aseptic loosening of both implanted stems, while five stems loosened twice. Twelve ASLs occurred after cemented stem fixation (femoral *n* = 8/8; tibial *n* = 4/6) while two ASLs occurred after cementless fixation (tibia *n* = 2/6).

In the femur, the metaphyseal distal femur was affected in five cases (*n* = 5/8). Two diaphyseal implants (DI) and three custom-made stems (CMS) were used. However, CMS mimicked the design of DI except for shorter stem length due to limited remaining bone stock. Two ASLs in the proximal metadiaphyseal femur occurred after implantation of tibial stems, one after CMS implantation mimicking DI.

In the tibia, three ASLs occurred in the metaphyseal distal tibia. All ASLs occurred using CMS mimicking DI stems regardless of cementation (cemented *n* = 2; cementless *n* = 1). Three ASLs occurred in the metadiaphyseal (*n* = 2) and metaphyseal (*n* = 1) proximal tibia. All those ASLs occurred using DI stems.

The metaphyseal region proved to be most vulnerable for ASL with a rate of 39.1% (*n* = 9/23; femoral *n* = 5; tibial *n* = 4). In meta-diaphyseal fixations, ASL occurred in five patients (31.3%; *n* = 5/16; femoral *n* = 3; tibial *n* = 2). In contrast, no stem loosening was seen in diaphyseal (*n* = 12) and epiphyseal (*n* = 5) sites.

**Fig. 10:**
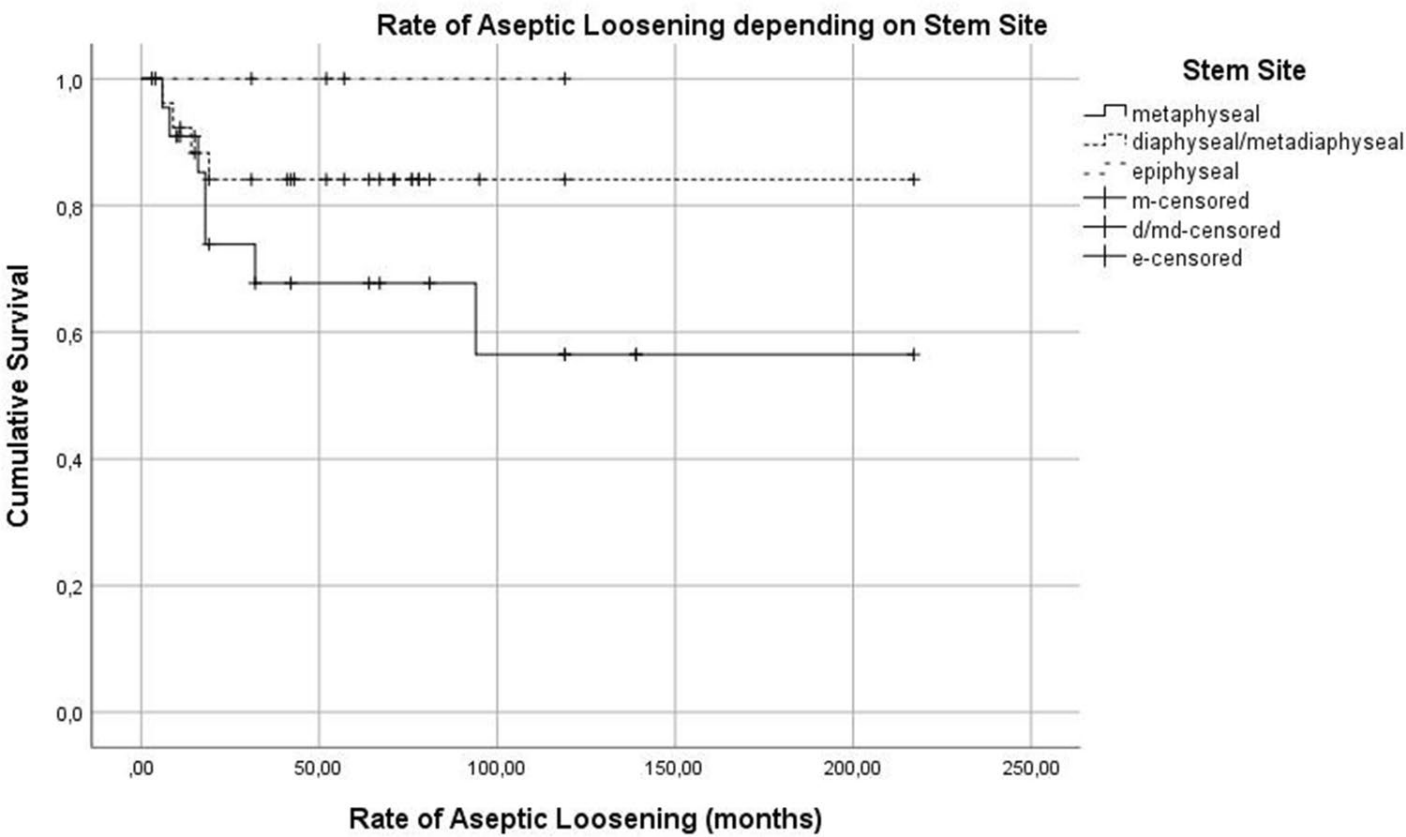
Kaplan Meier Curve—rate of aseptic loosening depending on stem site

**Fig. 11:**
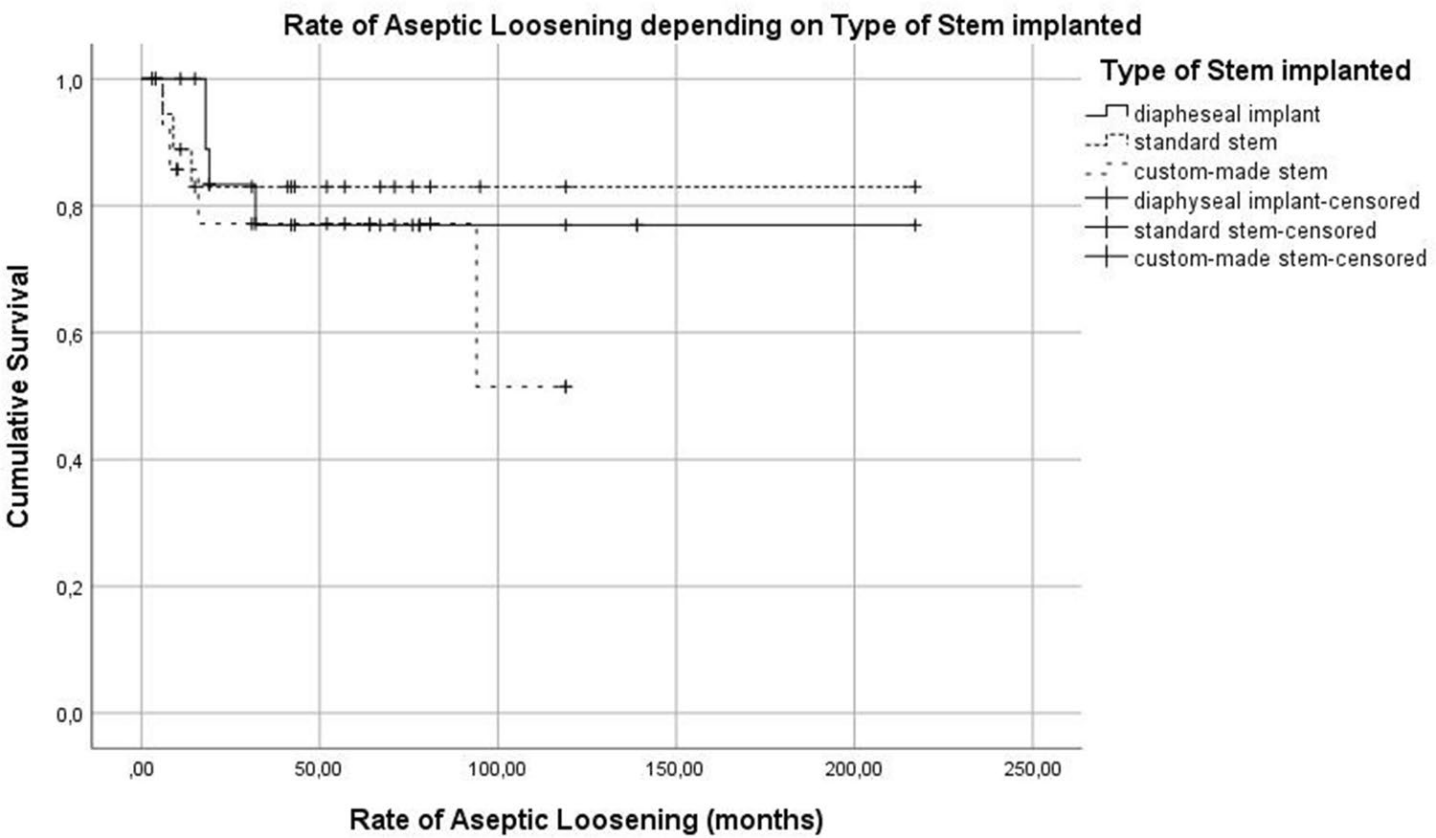
Kaplan Meier Curve—rate of aseptic loosening depending on stem implanted

**Fig. 12:**
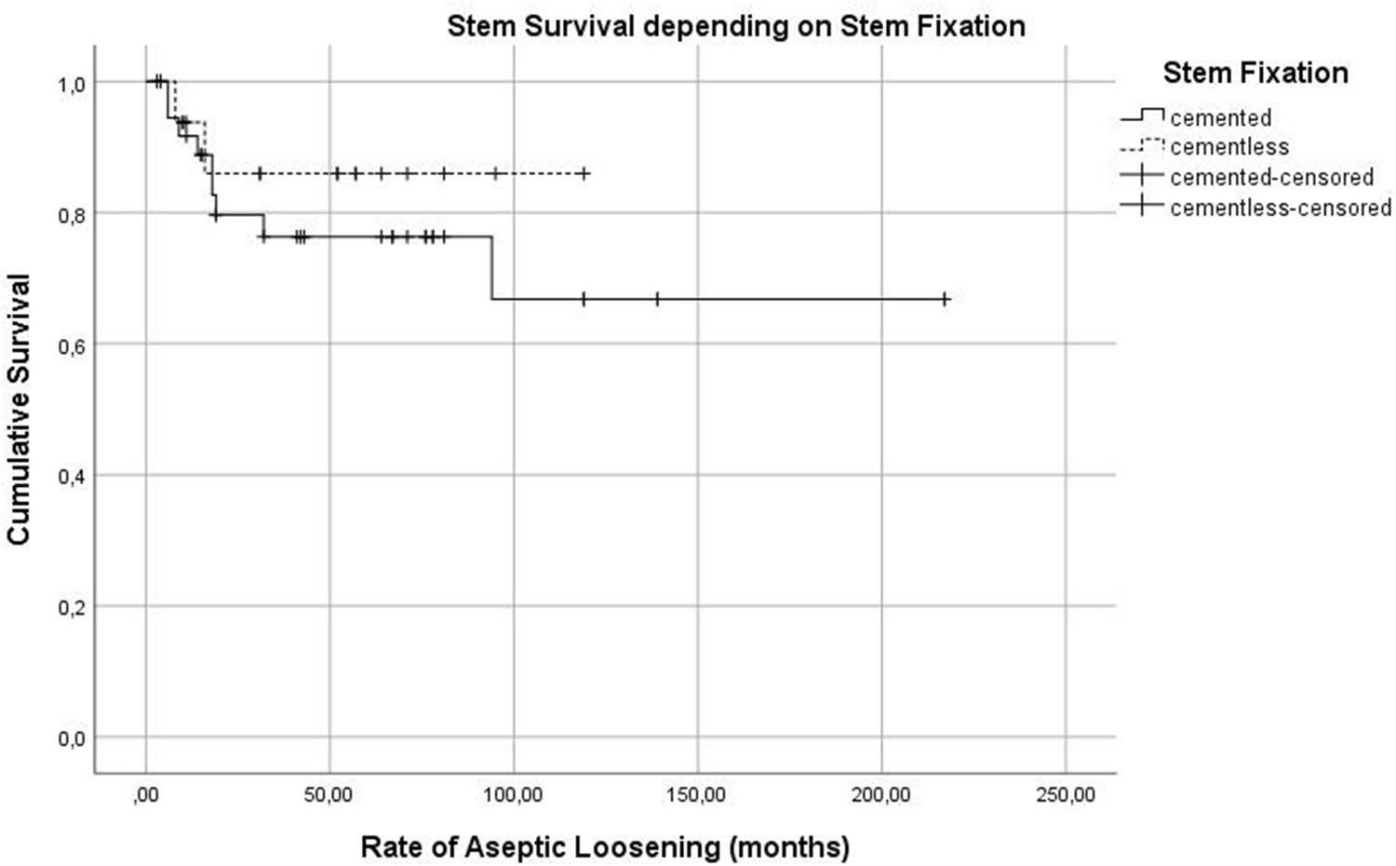
Kaplan Meier Curve—stem survival depending on stem fixation

The rate of ASL depending on stem site can be seen in Fig. 10. The influence of the stem design and cemented or cementless implantation technique can be seen in Fig. 11 and 12. Type of stem fixation (*p* = 0.453), stem design (*p* = 0.678) or implantation site (*p* = 0.244) was not statistically significant for the event of aseptic loosening according to the Kaplan Meier estimation calculated using a Cox regression model.

Despite its high frequency, ASL caused reconstruction failure in four patients only (14.3%). Patient 2 in the tibia group was amputated after two events of ASL and persistent pain with significant functional restrictions. Three other patients were converted using osteoarticular distal femur reconstructions after ASL of the distal femur. A more detailed overview of revision operations is presented in Tables 3 and 4.

#### Type 3—Structural failure

In one patient (3.6%) a traumatic periprosthetic fracture occurred 15 months after the initial operation. Due to multiple fracture fragments, the intercalary reconstruction had to be abandoned. The patient underwent a conversion operation and was reconstructed using an osteoarticular proximal femur replacement.

### Non-mechanical complications

#### Type 4—Periprosthetic infection

Implant-associated infections occurred in three patients (10.7%; femoral *n* = 1 and tibial *n* = 2). One patient with a femoral reconstruction was treated with a two-stage revision concept 5 months after the initial operation. This patient died of disease 67 months after replantation without any further revision procedures. Poor soft tissue coverage and a therapy-resistant infection in two patients with tibial reconstructions led to below-knee amputation in one patient. The other patient continues to refuse amputation despite our recommendation.

#### Type 5—Local recurrence

Local recurrence (14.3%; *n* = 4) resulted in amputation in three patients, while the reconstruction was intact at that time.

Overall reconstruction survival was 42.9% (*n* = 12) at a mean follow-up of 65 months. However, six patients died of disease (DOD) with intact implants after a mean time of 41 months after the operation, which negatively affects the overall implant survival (overall reconstruction survival disregarding DOD patients: 64.3%; *n* = 18).

Overall limb survival was 72.7% (*n* = 16/22). Limb salvage failed in six patients (27.3%). Amputation was necessary due to local recurrence in three cases, mechanical failure and infection in one case each. One patient had knee disarticulation for compartment syndrome without failure of the femoral segmental prosthesis. Limb survival was 100% for six patients at the time they died of the disease.

Prior operations before implantation of a segmental prosthesis did not have a statistically significant impact on reconstruction failure (*p* = 1.000) or limb loss (*p* = 0.119). Tumor size, reconstruction length and radiation therapy did not have a statistically significant impact on reconstruction failure (*p* = 0.954; *p* = 0.488; *p* = 0.593) or rate of revision operations (*p* = 0.671; *p* = 0.903; *p* = 0.831).

## Discussion

This study presents 28 patients treated by intercalary megaendoprosthetic reconstruction and implantation of a total of fifty-six stems in the lower limb. Overall limb and reconstruction survival were 72.7% and 64.3% (the last excluding DOD patients). Despite acceptable rates of reconstruction and limb salvage in this study, intercalary reconstructions of the lower limb remain a surgically demanding procedure. Both biological and endoprosthetic reconstructions are associated with high rates of complications and reconstruction failure in literature while providing similar results in terms of oncological outcome [5, 9–11].

Biological reconstructions, such as vascularized fibula autografts are more likely used in younger patients offering a chance of complete biological incorporation while restoring the bone defect [4, 11]. In terms of longevity and overall outcome, they are superior to sole allograft reconstructions. Bone healing at the docking sites in patients treated with chemotherapy and/or local radiotherapy is delayed or may be compromised. Long periods of non- or partial weight bearing are regularly needed to achieve bone ingrowth of the fibula autograft. Mankin et al. report 195 patients who received intercalary allografts after bone tumor resections. 13% (*n* = 22) of these ultimately failed due to either patient death of local recurrence, amputation or removal of graft due to recurrence or complication. Infection (*n* = 82; 11%), fracture (*n* = 140; 19%), non-union (*n* = 122; 17%) and unstable joint (*n* = 28; 6%) were observed as allograft complications in 718 procedures including but not limited to intercalary reconstructions and various implantation sites [12]. Aponte Tinao et al. also present eighty-three patients who were treated by intercalary femur segmental allograft reconstruction. Implant survivorship was 85% at 5 years and 76% at 10 years. Reconstruction failure was observed in 18.1% (*n* = 15) of cases, due to infection (*n* = 1), local recurrence (*n* = 1) and fracture (*n* = 13). Non-union was observed in 13% of docking sites (*n* = 22) and more common in the diaphysis (19%) than the metaphysis (*n* = 3%). Fixation by nail led to nonunion more commonly (28%) than plating (15%) [13]. Other authors in literature also report that use of solid allografts or allograft/fibular autograft compositions may offer primary stability and early full weight bearing but presents with high rates of non-union and secondary implant failure in as much as 60% of cases [1, 14].

Biewener et al. provide data on compound intramedullary nailing and bridging of the defect with a porous polymethylmetacrylate spacer as an alternate reconstruction method. They report treatment of 13 patients with that technique and found mechanical failure in one case only. Despite a low rate of complications observed, they concede that this technique may show promise as an interim solution during chemotherapy before permanent biological reconstruction. But as a definite reconstruction, it should remain limited to patients with poor prognosis and in elderly patients when callus distraction seems infeasible [15].

A high rate of complications of biological reconstructions notwithstanding, endoprosthetic reconstructions remain less popular in segmental reconstruction. Reports of early implant failure seem to outweigh their main advantages of lack of donor site morbidity, immediate primary stability and early full weight bearing [1, 2]. Yet, Sewell et al. present a series of eighteen patients with tibial reconstructions and an estimated 10-year implant survival of 63% [16], which is comparable to other series [1, 17] and indicating a moderately low complication rate. Major complications such as periprosthetic fractures, infections or implant failure are described to occur in less than 10% each [16]. Liu et al. report an absence of prosthetic-related complications in their retrospective analysis of 12 patients treated with intercalary reconstruction of the lower extremity at a mean follow-up of 22.5 months [18]. Lun et al. also report a favorable outcome for intercalary prostheses (*n* = 16) compared to segmental allograft (*n* = 18) in terms of overall complications (18.8% vs. 66.7%), implant-related complications (12.5% vs. 55.6%) and reoperation rate (12.5% vs. 38.9%) at a short-term follow-up. Time to full weight bearing and hospital stay were also shorter in the segmental prosthesis group [19]. Even limb salvage by implanting segmental prostheses after failed biological reconstructions has been reported with promising results [20]. One (3.6%) periprosthetic fracture and three infections (10.7%) in the presented series reflect these satisfactory results. In particular, the infection rate is lower compared to that of other reconstructions involving a joint replacement, which is published to occur in as many as 43% of cases in the lower extremity [21].

In this study, aseptic loosening was the most frequent complication observed at a rate of 53.8% (*n* = 14). Despite such a high rate of ASL observed, reconstruction failure caused by ASL remained fairly low at 14.3% (*n* = 4). Our ASL rate of 25% (*n* = 14/56 stems) compares with Abudu et al.’s findings who present data on eighteen patients reconstructed with custom-made diaphyseal implants and shorter than conventional stems and observe a necessity for surgical intervention in 33% of cases (*n* = 6/18) due to ASL [9].

Analyzing the cause of ASL, our data imply that meta-diaphyseal and metaphyseal stem site, as well as the configuration of standard stems are anatomically and biomechanically incompatible and primary as well as long-term stability cannot be sufficiently achieved. In 85.7% (*n* = 12/14) of cases, ASL occurred after cemented stem fixation in this collective. Metaphyseal (39.1%; *n* = 9/23) and metadiaphyseal (31.3%; *n* = 5/16) implantation sites were most commonly affected by ASL, while it was absent in diaphyseal and epiphyseal sites. All stem failures in the proximal and distal femur (*n* = 8) and tibia (*n* = 6) occurred in metadiaphyseal and metaphyseal sites, using either cemented tibial stems, diaphyseal implants or custom-made stems mimicking diaphyseal implants except for stem width and length.

Fuchs et al. are in agreement with this observation, defining a remnant bone stock of less than 5 cm in length as a contraindication for the use of segmental prostheses using standard stems [2]. An in vitro study by Bischel et al. also found indications that stem properties may vary depending on implantation site and publishing that tapered stem designs seem to be favorable in larger defects whereas the hexagonal may be advantageous in defects located more distally [22].

Tedesco et al. report six patients who received customized anchor plugs for short-segment fixation with a double compressive osteointegration intercalary implant at a mean follow-up of 39 months. They report stable fixation was achieved in all reconstructions, with the shortest remaining stem length measuring 3.7 cm. While they report no cases of aseptic loosening, three mechanical failures had to undergo revision surgery. Yet, secondary adjacent joint replacement and amputation were avoided in those cases [23].

Even though stem revisions did not cause implant-associated infections in the presented collective (0%), one has to bear in mind that every revision procedure carries a risk of infection which is described to be as high as 12% for megaprostheses in tumor patients [24, 25].

While this study did not find that additional interlocking screws led to better fixation rates after cemented stem fixation, Vorys et al. reported that locking fixation resulted in a stronger construct with increased cycles to failure compared to non-locking fixation of a segmental scaffold [26].

The study by Tedesco et al. [23], the findings presented in this patient collective, as well as the case series published by Guder et al. [7], seem to support that adaptations of stem design depending on implantation site might solve the high rates of aseptic loosening observed after segmental endoprosthetic reconstruction.

Therefore, the findings of this study—indicating that adaptation of stem design may reduce the high rate of aseptic loosening, and thus implant failure, in segmental endoprosthetic reconstructions—have an impact on future patient counseling and planning segmental reconstructions in our clinical practice: the Buxtehude stem (see Stem properties ad 1.) and hollow hexagonal stem designs show prospect in improving primary and long-term stability rates compared to standard stems in metaphyseal, metadiaphyseal and epiphyseal sites and should be considered depending on remaining bone stock. Interlocking screws and implant flaps modeled anatomically to increase the contact surface on the outside of remaining bone (see Fig. 6c) also seem feasible and should be considered for challenging stem sites such as the distal femur and distal tibia for improving stem fixation. Cementless fixation relying on osteointegration is preferred when feasible (i.e. patient age, comorbidities etc.). Collection of patient and treatment data of patients indicated and undergoing reconstruction using these novel stem designs has already commenced prospectively to be analyzed and improve evidence of the trends observed in this study in the future.

## Conclusions

Segmental megaendoprosthetic reconstructions are a feasible option of reconstructing intercalary bone defects of the lower limb. If increased complication rates should decrease due to introduction of novel, more biomechanically and anatomically suitable stem designs in larger patient collectives in future, indications for this procedure might extend to a younger collective with localized disease and a good overall prognosis as a standard procedure, rather than remain reserved for elderly patients with a limited life-expectancy.

### Limitations

We acknowledge several limitations of our study. Due to the retrospective study design, a small patient collective, eight different stem designs implanted and lack of a control group, it is difficult to draw objective conclusions. Also, the follow-up period may be too short and we cannot rule out the possibility of complications occurring in the long term that have not been detected yet. Larger-scale studies with a more homogenous patient collective are necessary to confirm the findings of this analysis and improve evidence-based decision-making.
